# Thermal transport in kinked nanowires through simulation

**DOI:** 10.3762/bjnano.14.49

**Published:** 2023-05-15

**Authors:** Alexander N Robillard, Graham W Gibson, Ralf Meyer

**Affiliations:** 1 Bharti School of Engineering and Computer Science, Laurentian University, Sudbury P3E 2C6, Canada

**Keywords:** ballistic transport, kinked nanowire, molecular dynamics, phonon Monte Carlo, thermal transport

## Abstract

The thermal conductance of nanowires is an oft-explored quantity, but its dependence on the nanowire shape is not completely understood. The behaviour of the conductance is examined as kinks of varying angular intensity are included into nanowires. The effects on thermal transport are evaluated through molecular dynamics simulations, phonon Monte Carlo simulations and classical solutions of the Fourier equation. A detailed look is taken at the nature of heat flux within said systems. The effects of the kink angle are found to be complex, influenced by multiple factors including crystal orientation, details of transport modelling, and the ratio of mean free path to characteristic system lengths. The effect of varying phonon reflection specularity on the heat flux is also examined. It is found that, in general, the flow of heat through systems simulated through phonon Monte Carlo methods is concentrated into a channel smaller than the wire dimensions, while this is not the case in the classical solutions of the Fourier model.

## Introduction

The thermal conductivity of semiconductor nanostructures is of great interest because of potential applications in a wide variety of fields, such as thermal control, communications, and many others [1–5]. In many nanoscale systems, thermal transport cannot be simply described as it would be at larger scales [6]. At such scales, the carriers of energy (such as phonons) have finite transit lengths that are no longer negligible compared to the system dimensions. Systems where such transport is important are said to have significant ballistic transport compared to the classical scenario, that is, diffusive transport. Ballistic transport can be impacted by features of the system, such as surfaces, edges, defects, and inclusions [7–9]. Consequently, the effect of system design on nanoscale transport is particularly intriguing and has lead to the investigation of unique structures [10–11] in an attempt to better understand and develop manufactured devices. The introduction of additional surfaces and the reduction of direct paths through a system can force additional scattering of phonons at boundaries and cause interesting effects due to phonon reflections [12–14]. These effects are typically not seen at the macroscopic scale, but instead reflect the sophisticated nature of transport and finite sizes at the nano- and mesoscales.

Recently, there have been investigations of kinked, serpentine, or chicaned nanowires, that is, wires with one or more right angles preventing a straight path along the wire between two temperature-controlled areas. While there are studies devoted to simulation [15–16], several examples of synthesis for these nanowires and nanosystems have been achieved [17–20]. We have also seen investigations into corrugated nanowires, where a jagged or sinusoidal pattern is inscribed into the edges of the wire [21–22].

In such engineered systems, it is common that there is a decrease in thermal conductance. There is a lack of free paths for unfettered ballistic phonon transit, suppressing the contribution to thermal transport. Heron et al. [23] found that nanowires with square serpentines with dimensions of a few hundred nanometers exhibit reductions in thermal conductivity of the order of 20–40%. Zhang et al. found that, in boron carbide nanowires [24], the thermal resistance of a kink can be 30 times larger than that of the straight portion of a wire. Anufriev et al. [22] investigated serpentine wires over a temperature range from 4–300 K and likewise found significant reduction in thermal transport, though this becomes less noticeable at higher temperatures. This trend couples with system dimensions, where shorter systems exhibit more noticeable effects than longer ones at the same temperature. Overall, both simulation and experiment find significant reductions in thermal transport when bends or kinks are introduced, though the dependencies for this are myriad and somewhat complicated to understand.

In this work we explore the effect of kinks where the angle is different from the 90° bend commonly used in other studies. We construct nanoscale systems with kinks of varying angles (ranging from 0° to about 70°) in order to examine the effect of disruption of ballistic paths in varying degrees on thermal transport. By varying the kinks, it is possible to better understand the nuances of blocking [23] or partially blocking phonon transport. Intuitively, we should expect a reduction in thermal transport with increased angle, as a steeper angling of the wire should result in reduced quantities of unrestricted ballistic transport. Gradually increasing the angle effectively reduces the quantity of unobstructed line of sight (LoS) paths from one end of a nanowire to the other. We use molecular dynamics (MD) [25] to study an atomistic approach to thermal transport, as well as a phonon Monte Carlo (PMC) simulation [26–29]. MD results will additionally be investigated for effects of lattice orientation in the wires and compared to a simple theoretical investigation of the LoS in these wires.

The MD and PMC results cover different views of thermal transport: MD is more sensitive to characteristics of the lattice, while PMC ignores lattice properties and provides insight to phonon behaviour. MD and PMC results are compared to a theoretical solution of classical heat transport using the Fourier equation. Combining the three methods (MD, PMC, and the theoretical solution of the Fourier equation), we find that the thermal conductance of a kinked nanowire varies significantly with the kink angle, but that the trend is not a simple monotonic function of the kink angle. Furthermore, heat flux data yielded by PMC and classical solutions provides detailed insight into how heat flows through kinked systems. In these kinked systems we will identify familiar “corner cutting” and “shadowing effects” [22] as well as heat channelling effects [30] similar to those seen in 90° serpentine systems. Combining MD, PMC, and theoretical solutions of the heat equation serves to better bridge the gap between heat transport phenomena at the macro- and the microscale and shows that multiple factors are significant in kinked systems beyond disruption of ballistic paths, including lattice orientation and phonon reflections.

## Results and Discussion

**Figure 1:**
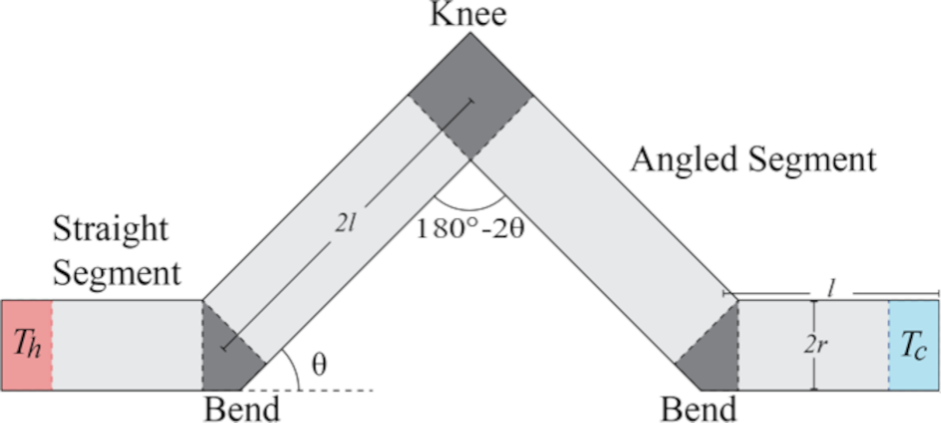
Schematic of kinked wire with nomenclature. The use of the length 2*l* is a typical example. Note the relation between the angle at the bend (θ) and the angle at the knee (180° − 2θ).

To understand the behaviour of the systems in question, we must elaborate on their geometry and design. The kinked wire systems consist of a general construction shown in Figure 1. At each end of the wire is a straight segment of length *l*, with a thermostatted region placed at each end. These straight segments provide a region for the effects of the thermostat on local heat transport to be reduced before reaching later portions of the wire. Connecting the two straight segments are two angled segments of lengths of some multiple of *l*. The radius of the wire is denoted *r*. The joining segments in the wires are referred to as the knee, connecting the two angled segments, and the bends, each of which is a connection between a straight segment and an angled segment. The angle of the angled segments with respect to the initial direction of the straight segments (at the bend) is denoted θ and is called the kink angle. The angle between the two angled segments (at the knee) is consequently 180° minus 2θ.

### Thermal conductance of kinked nanowires

The thermal conductance, *C*, is one of the most important basic properties of a nanoscale system. It is obtained from the total heat flowing through the system per unit time, *P* divided by temperature difference Δ*T* throughout the system:


[1] $$C=\frac{P}{\Delta T}.$$

#### Molecular dynamics

**Figure 2:**
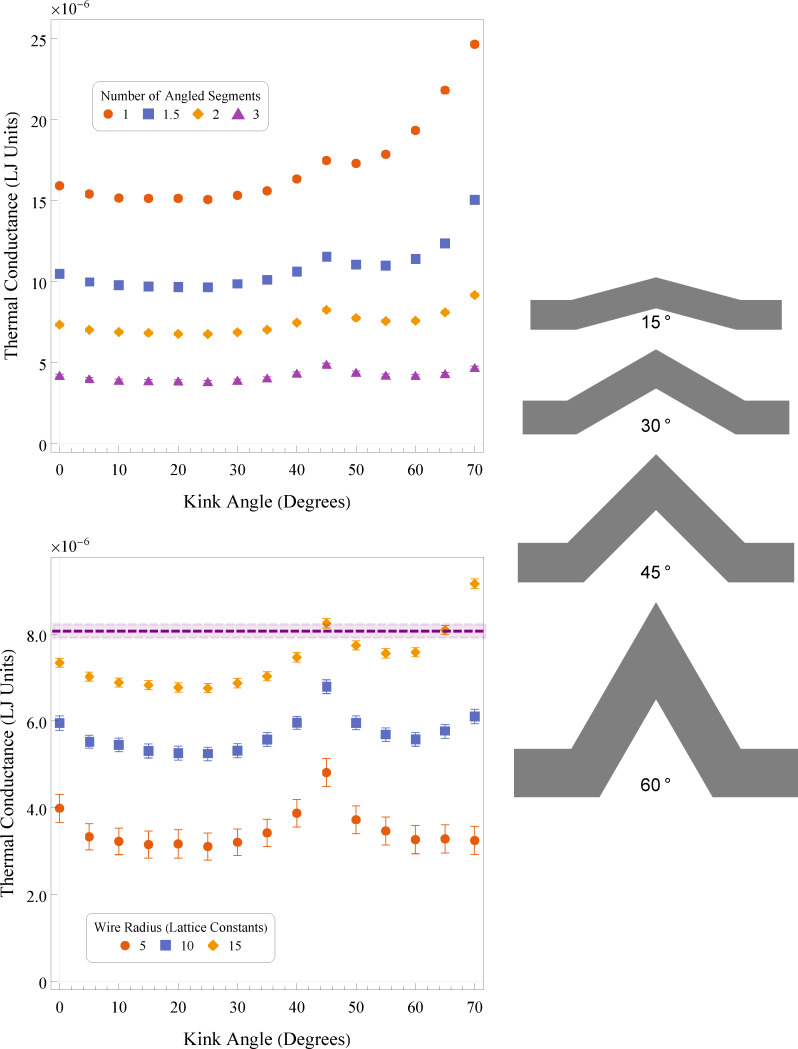
Top: thermal conductance of kinked nanowires with varying base segment length. Bottom: thermal conductance of MD-simulated nanowires for varying kink angles. Radii of wires varied from 5 to 15 lattice constants. Error bars are 1 standard error. The purple dashed line indicates the thermal conductance of a straight nanowire with similar length, a radius of 10 lattice constants, and the lattice orientation along the [110] direction. The shaded purple area indicates 1 standard error from the dashed line. Right: 2D reference sketches for a system with various bend kink angles with *r*/*l* = 1/3 and two lengths of *l* in each angled segment. This roughly corresponds to the blue squares data set.

In Figure 2 we show the thermal conductance of kinked nanowires in MD simulations as they vary with kink angle. The cylindrical nanowires were simulated using a Lennard-Jones potential and built on an FCC lattice. Dimensionless Lennard-Jones units are used throughout this work. Further details can be found in the Methodology section. The value of *l* is 30 lattice constants, with *r*/*l* = 1/3. The kink angles range from 0° to 70°. The upper panel shows the thermal conductance for varying angled segment lengths. The angled portions of the wire have values of 1, 1.5, 2, or 3 times the values of *l*, while the straight segments remain constant. This means that the shortest systems have a total path length of 120 lattice constants, and the longest a path length of about 420 lattice constants. The smallest MD systems have radii of 5 lattice constants, and the largest of 15 lattice constants. The lower panel of Figure 2 shows the trend with variation in radius.

The general trends of the thermal conductance with system dimensions agree with expectations from simple classical theory and with the general trends at the nanoscale [31–34]. As the segment length, and consequently the system length, are increased, the overall thermal conductance of the system decreases, shown by the decreasing values of the curves in the upper panel of Figure 2. Longer systems lead to longer path lengths for thermal transport, thus reducing the conductance (or equivalently, increasing the thermal resistance) for the same temperature difference. Works on serpentine nanowires show similar behaviour [22], with thermal conductivity asymptotically approaching a constant (which is analogous to an inverse proportionality for thermal conductance).

Similarly, an increase in wire radius for a constant segment length is expected to result in an increase in thermal conductance as more cross-sectional area results in more heat transport. This is seen in the lower panel as the values of the curves increase with increasing radius.

It is known that nanoscale simulations can result in unexpected trends of thermal transport with system dimensions [35]. Since the variation in length and radius is over a small range, we do not see any anomalous trends in the thermal conductance.

All the systems presented have broadly similar trends up to about 55° or 60° of kink angle, independent of their segment lengths or radii. The thermal conductance has a clear dependence on the kink angle. It decreases until approximately 15° to 20° of kink angle before reaching an inflection point and then increasing to a peak around 45°. Beyond 45° the thermal conductance begins to decrease again until 55° or so, before increasing dramatically. The large increase at very high kink angles is due to the anomalous geometry in the systems. When the angle becomes steep, the surface area at the knee begins to grow as the two angled segments begin to clip into each other. This results in an unusually large cross section at the knee and also aggravates potential sintering problems. This effect is related to wire length and radii as these factors determine when the wire will begin to merge onto itself at the knee.

It is notable that the scale of the effects seems to be less pronounced for systems with either long segment lengths or smaller wire radii. The impact of segment length and wire radius is similar, as both control the ratio *r*/*l*. The ratio between the radius and the segment length partially dictates the quantity of phonons that can travel large distances without scattering off a surface. With a small ratio, we must take into account the nature of phonon scattering and reflection into our model of heat transport. This idea ties in to the familiar Knudsen number, Kn = λ/*L*, with λ being some transport mean free path and *L* being a measurement of the typical transport length through the system. In the case of the kinked nanowires, this transport length is more likely the wire radius, *r*, than the total length of the system. In fluid mechanics, a large Knudsen number (near or greater than 1) typically indicates the unsuitability of a continuum approach. In the case of phonon transport, we often take this as an indicator of strong ballistic transport over diffusive modelling.

Through the design of these systems, two factors control the limitation of the mean free path for heat transport by inducing phonon collisions with a surface. The first is the ratio of the radius to the system length. It is common in low-dimensional systems to find unimpeded ballistic heat transport being suppressed in systems with small radii or long lengths. In such scenarios, few ballistic transit paths make it through without scattering against a system boundary; the longer or narrower the system, the fewer such paths. Given the sizes of the systems simulated here, these effects are likely to be significant. Contributions to thermal conductivity in simulated systems are significant for mean free paths of the order of 10 lattice constants or more [36]. Second, in kinked systems specifically, the kinks further limit those paths through a form of phonon blocking [23]. Introducing a bend in the wire provides a barrier to direct ballistic transit, as there are fewer and fewer straight paths through the system as the bend angle increases.

Theoretical insight on the effects of this barrier can be obtained through a simple analysis of the LoS. Park et al. [30] categorized systems into blocked LoS systems, where there are no direct paths for transit from one end to the other, and continuous (or unblocked) LoS systems, where there are direct lines through the system that do not intersect any walls or boundaries. This classification was used to examine the effects of LoS reduction on thermal transport. In the kinked wire systems we discuss here, it is possible to approximately quantify the amount of unblocked LoS by determining the area of overlap of two circles, one at the bend of a kinked wire and the other at the knee. The overlap area of the two circles is related to the number of lines one can trace through the wire from one end to the other without intersecting a surface (*A*_overlap_ ∼ LoS). Only one bend needs be considered due to symmetry. For a kinked wire with radius *r*, segment length *l*, and bend kink angle θ, as previously described, the overlap area (here as a fraction of *l*^2^ for convenience) is given by:


[2] $$\begin{array}{c} \frac{A_{\text{overlap}}}{l^{2}}=-\sin\left(\theta \right)\sqrt{\frac{4r^{2}}{l^{2}}-4\sin^{2}\left(\theta \right)}\\ -\frac{2r^{2}\tan^{-1}\left(\frac{2\sin\left(\theta \right)}{\sqrt{\frac{4r^{2}}{l^{2}}-4\sin^{2}\left(\theta \right)}}\right)}{l^{2}}+\frac{\pi r^{2}}{l^{2}}. \end{array}$$

**Figure 3:**
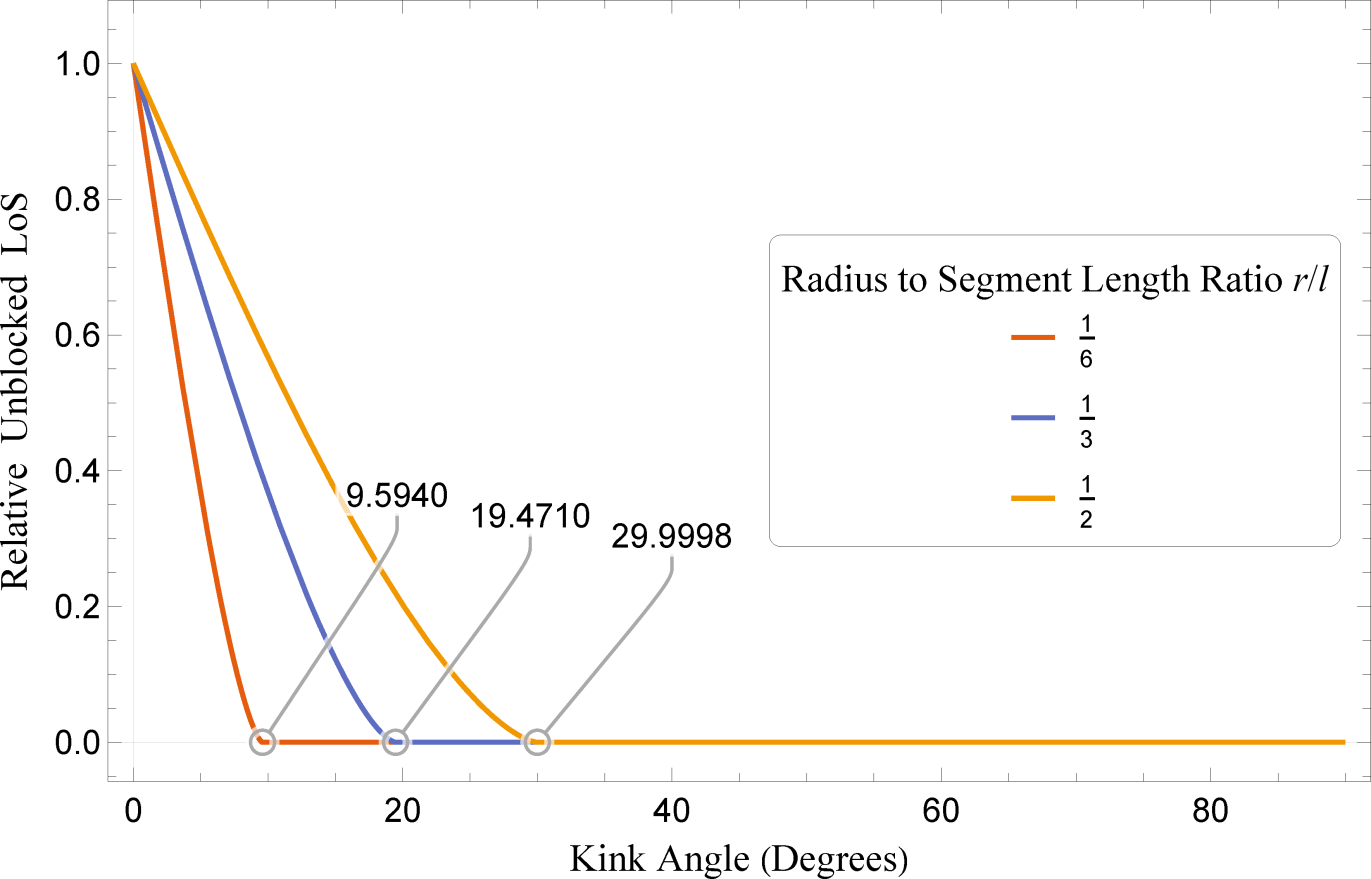
Relative LoS through kinked wires as a function of both kink angle and radius to segment length ratio. Values are expressed as a ratio of total LoS area to maximum theoretical, and threshold angles where LoS = 0 are highlighted.

Figure 3 shows the unblocked LoS of different systems as a fraction of the maximum possible value, which occurs in a perfectly straight wire. These coloured curves correspond with the geometries of the equivalently coloured data points in the lower graph of Figure 2. Also highlighted are the points at which the curves reach 0 (i.e., the point at which the LoS is fully blocked). We see that classifying systems as having fully blocked (LoS ≤ 0) or partially unblocked (LoS *>* 0) LoS depends on the system geometry. For systems with a very small *r*/*l*, angles as low as 10° can be considered as having fully blocked LoS, while for larger *r*/*l* values, LoS can be partially unblocked up to values just below 30°.

The trend of LoS shares one similarity with the conductance data: For low kink angles it decreases, up until the point where LoS is fully blocked. Otherwise, these curves and the conductance data are dissimilar. There appears to be no significant changes in where the local minimum conductance occurs with changing *r*/*l*, suggesting that LoS has a rather minimal effect once the kink angles start to become significant. This would seem to conflict with results in labyrinthine nanowires [30], but in the case of the kinked wires studied here, with only one kink, gradually reducing the LoS does not result in a significant increase in the path length, as is common in square serpentine constructions. The kinked wires were specifically constructed to minimize the change in path length due to the change in kink angle.

We have shown that the thermal conductance of the wires does not necessarily align to predictions from LoS analysis. In addition, the conductance is not monotonically decreasing. To explain the increase in conductance for angles beyond 20°, we must consider that the thermal transport in a nanostructure may differ when travelling along orientations of a crystalline lattice different from the [100] direction. Though the thermal conductivity of a theoretical crystal with cubic symmetry is isotropic (and investigations of perfectly cubic systems have found such [37]), because of the limited dimensions of a nanostructure along certain crystal axes the contribution to thermal transport in the lattice can be anisotropic. In addition, anisotropic heat transport has been found in silicon nanosystems [38], where the thermal conductivity can vary based on the lattice orientation along the direction of transport. It is possible that this effect is related to how lattice orientation can affect surface scattering. Zhou, Chen and Hu showed that a [110] surface-oriented lattice has a significantly increased thermal conductivity compared to a [100] surface-oriented lattice [39].

There are then two competing factors here in the thermal conductance, namely the complex effects of the kink itself and the orientation of the lattice along those kinked segments. Since the wire is single crystalline, the main direction of heat transport along the angled segments will be along a lattice direction that corresponds to that angle. At 45°, the angled segments are oriented along the [110] direction of the crystalline lattice, which results in a significant increase in thermal transport. The dashed purple line in the lower panel of Figure 2 represents the thermal conductance of a straight [110] FCC wire with *r* = 10 lattice constants. We see that it is significantly increased compared to its equivalent (blue squares) data set. This is because this wire does not have a kink, but rather is perfectly straight.

**Figure 4:**
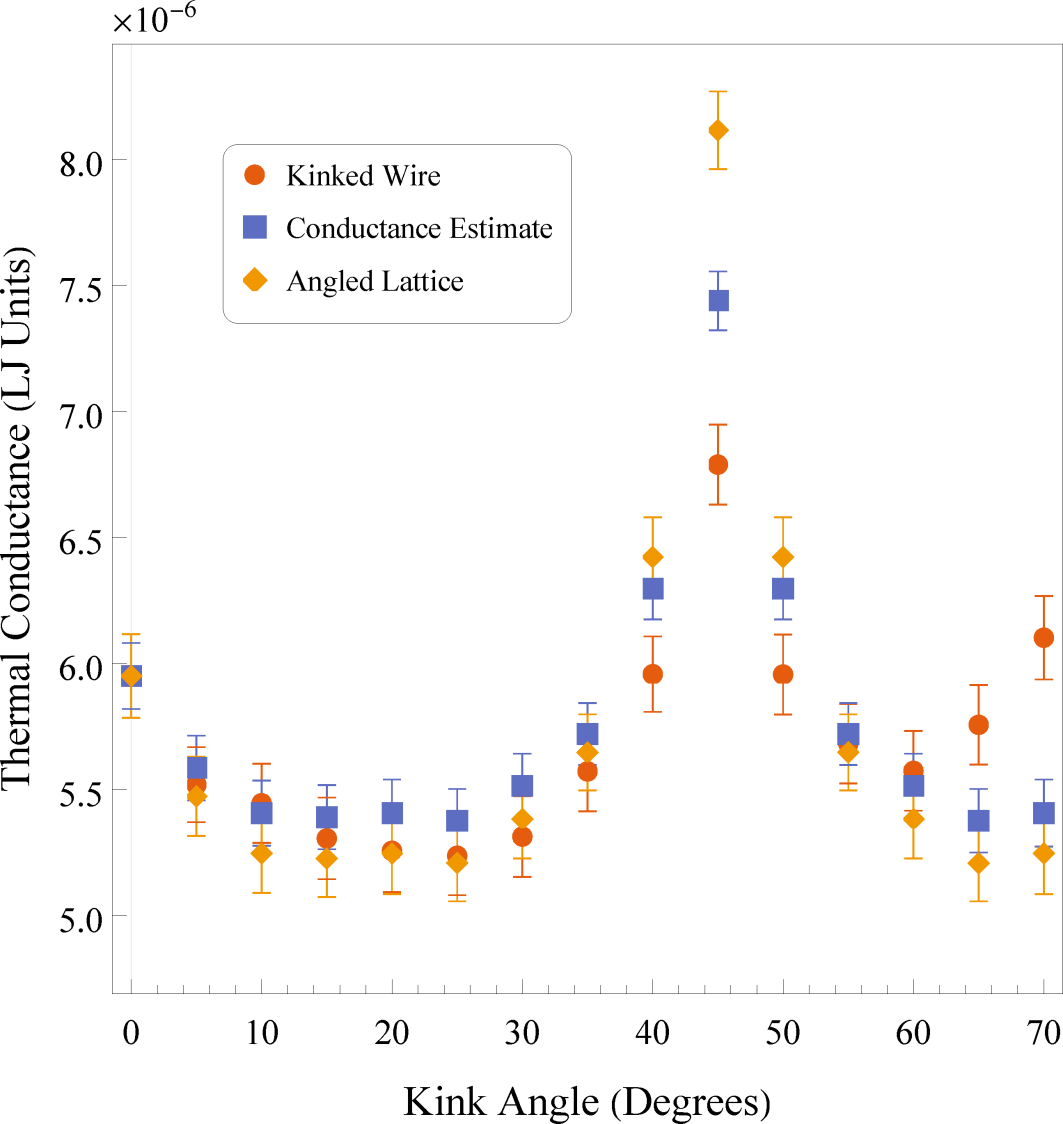
Comparison of thermal conductances for kinked wires, straight wires with angled lattices, and a series resistance estimate.

To better understand the effect of the kink itself versus the effects of lattice orientation, we have simulated a set of straight wires with *r* = 10 lattice constants where the lattice orientation is angled as to mimic the lattice orientation in the angled segments of a corresponding kinked wire. Using these results, it is possible, with proper weighting, to estimate the conductance of a kinked wire where only the lattice orientation matters. Figure 4 shows the previous kinked wire data, a set of data for the straight wires with angled lattice orientations, and the conductance estimate. From Figure 4, we see that overall the thermal conductance of the straight wires with angled lattices is quite high, while the conductance estimate is overall higher than the true kinked wire data. This implies that the lattice orientation is not the only important effect here, even though the effects of the angled lattice closely follow those of the kinked wires with varying angle. One would expect that the angles in the wire limit the quantity of ballistic phonons able to transit freely through the system without scattering off a surface, and the consistent disparity between the conductance estimate and the true kinked wires shows that this is a contributing phenomenon.

#### Phonon Monte Carlo and Fourier equation

We believed that a significant decrease in the thermal conductance of the wires would occur corresponding primarily to the reduced number of straight ballistic paths through the wire, or equivalently the LoS. It would instead appear that the changes in conductance in MD results are as much due to the angling of the lattice as the kink effects. In order to further investigate the effects of the kinks, we turn to phonon Monte Carlo simulations. PMC simulations usually treat the simulated material as isotropic. While this is typically seen as a missing component in PMC, in our case it provides a significant advantage: We can examine the thermal transport of phonons without including the effects of the lattice orientation. This is particularly useful for insight into applications where lattice orientation is less of a concern, such as in larger nanosystems.

2D nanowire systems constructed with a similar geometry to those in the MD simulations were simulated with PMC to examine thermal transport. The segment length *l* is set to 120 nm and the wire radius is 40 nm. The PMC simulates the transit of acoustic phonons through these 2D systems and factors in scattering with boundaries, but does not incorporate optical phonons. Results are compared to a numerical solution of the Fourier equation for the steady state. This will provide a theoretical method to contrast the nature of heat transport in a diffusive scenario to the phonon-based picture of PMC.

**Figure 5:**
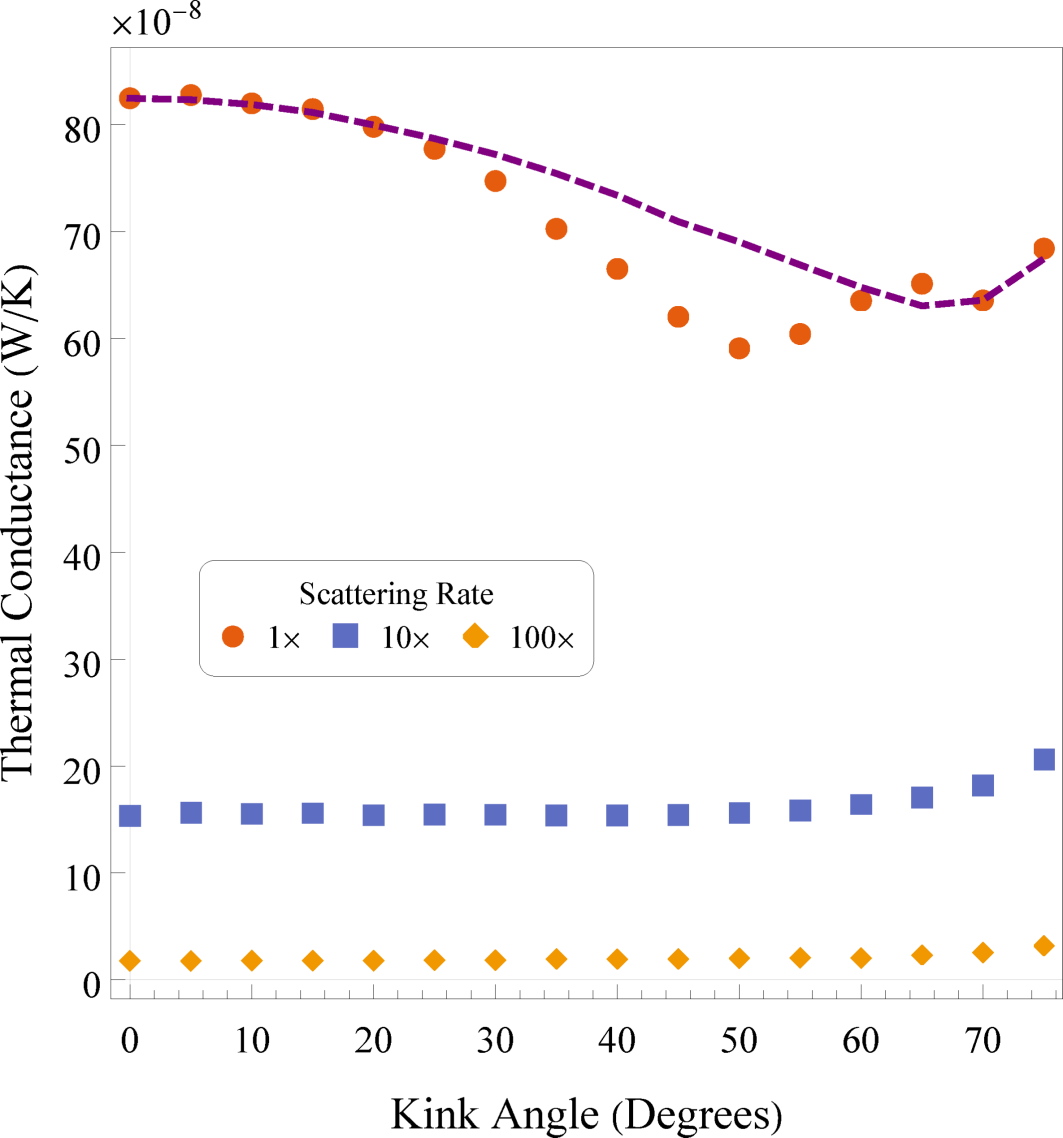
Thermal conductances calculated from 2D PMC simulations of kinked nanowires with varying kink angles. Three scattering rates are shown. The purple line shows the (rescaled) conductance yielded by the Fourier result.

Figure 5 presents the thermal conductance of systems against kink angle for three scattering rates. The three scattering rates (1×, 10×, and 100×) are used to trend the results of the PMC simulations to more and more diffusive cases. The 1× scattering rate corresponds to rates observed in silicon, while the higher rates represent an artificial case where ballistic transport is suppressed. Details on the methods used for calculation of scattering rates are elaborated in the Methodology section of this work. The thermal conductance of these 2D systems was extrapolated to 3D by assuming a square cross section for the wire. In addition, we have varied the scattering rate to allow us to see how much ballistic transport impacts thermal conductance in the systems. The dashed purple line in Figure 5 shows the mean system flux for the numerical solutions of the Fourier equation. Since the temperature difference is constant, this mean system flux is related to the thermal conductance by a constant and is scaled to match the PMC result for 0°. At 0° of kink angle, we have the least geometric effects occurring in the wire. Hence, we can expect some degree of similarity in behaviour between the purely diffusive solution of the Fourier equation and the ballistic scenario occurring in the PMC simulation.

In Figure 5 we see that the PMC thermal conductance decreases with increasing kink angle until about 50°. After this point, the conductance begins to increase again. As this is not due to lattice orientation, this effect must be due to the interactions of travelling phonons with the surfaces of the wire due to the kink angle. In the PMC simulation, phonons are reflected from a surface after collision based on their angle of incidence. As these travelling phonons have a chance to scatter because of the presence of other phonons, this tends to increase the distances phonons must travel to transit from one heat bath to the other. As the kink angle increases, more and more phonons scatter off a boundary surface at least once, and as the angle increases further, more of them are reflected multiple times in the wire. Interestingly, as the angle increases beyond around 50°, there is an increase. This may be due to a geometric effect, that is, there should be some angles, based on wire shape and kink angle, that are local minima for the number of reflections occurring for most paths. Cook and Varga [40] show that this can be the case in 1D nanowires, where kinks in general result in poor transmission, but there can be resonant states for certain geometries. Note that the trajectory of a phonon travelling straight out of the initial portions of the wire should deviate by an angle of 2θ as it collides with the inside part of the kink.

Comparing this data with the results from the solution of the Fourier equation, the initial few points seem to align quite well. The conductance slowly begins to decrease with increasing kink angle. As we increase the kink angle, we see that the PMC result begins to decrease more than the theoretical result from the Fourier equation. The reflective nature of the ballistic phonons causes them to more often take indirect paths through the system. The Fourier solution, in contrast, decreases rather smoothly through this region. When the kink angle approaches 60° or so, the conductances begin to agree again, right before anomalous geometric effects dominate (if one were to continue the dashed line, one would find that the curve increases dramatically as we approach the ill-defined 90° kink angle). The dip in conductance from the PMC simulations when compared to the solution of the Fourier equation may reflect geometric effects discussed earlier. As the phonons travel in a ballistic fashion, they can be more significantly affected by a large kink angle, leading to a steeper slope.

Artificially tuning the scattering rates allows us to investigate certain hypotheses. As the transport becomes less ballistic, the effects of the kink should be reduced overall. Looking to the 10× increased scattering rate, we see that much of the effects due to the kink seem to vanish, save for a slow increase with increasing kink angle. With the scattering rate becoming large, and consequently the mean free path of phonons becoming short, the effect of scattering off surfaces becomes negligible, and the heat flow becomes more diffusive (it is also reduced in magnitude due to overall reduced transport). In contrast, the trends in conductance seen in the previously discussed (1× PMC and solution to the Fourier equation) curves are not well observed in these curves. This may be due to the fact that the higher scattering rates here are not truly diffusive, and that the scale of the systems is such that the ballistic effects seen at a lower scattering rate (equivalently, a larger mean free path) are not prevalent here.

In many cases, the slow increase with high kink angles might come from a “corner cutting” behaviour. As the kink angle increases, because the wire has a finite thickness, there are small “shortcuts” that the heat flow can use to reduce its effective path length, much like a race-car driver taking the inside corners of a series of curves. This corner cutting has been seen and discussed previously in the context of right-angled serpentine nanowires [20,22,30].

To reiterate a point mentioned earlier, in all the previous figures (for all simulation types), we have seen a notable jump in the last couple of data points. Because the kinked wires in the 3D MD simulations are constructed by intersecting two cylinders at an angle of θ, when this angle becomes large (typically above 60°) the cross-sectional area of the intersection becomes significant and this change in area affects the result both by artificially amplifying corner cutting and by allowing for a larger surface through which heat can flow. The 2D systems simulated here were constructed to reflect this fact and have, thus, a wider cross section at the knee. In the extreme cases towards 90° kink angle, the straight portions of the wire nearly contact each other and the result is a rather short, nearly straight wire with a large wire attached perpendicularly at the middle. As such, results for values above 65° of kink should be taken with a grain of salt, particularly in the MD simulations.

### Heat flux field

In systems with complex geometry, the nuances of heat transfer beyond broad thermal conductance can be understood by examining the heat flux throughout the system. By investigating the flux through kinked systems, it is possible to identify underlying phenomena such as corner cutting and reflections. The existence of these effects can be indicators of the importance of nanoscale heat transport phenomena in the systems in question. In addition, these phenomena can result in non-uniform flows of heat through systems, which may significantly affect functionality. Using colour maps and 2D vector plots, it is possible to take an in-depth look at heat transport in the kinked nanowires.

**Figure 6:**
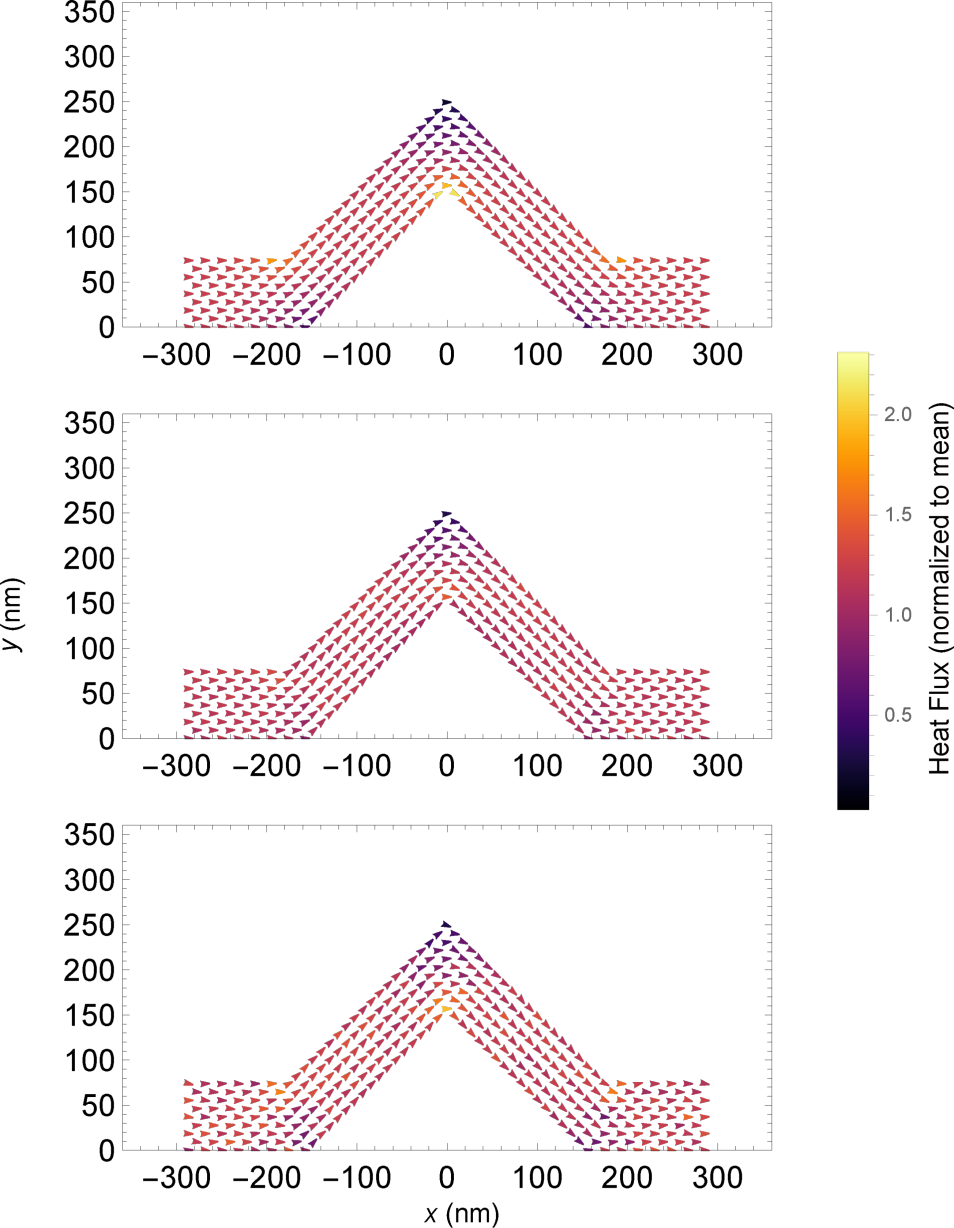
2D vector plots of heat flux in 45° kinked wires. The colour mapping indicates the value of the heat flux, normalized by the mean flux, through the system, yielding a unitless flux. Top: Fourier steady-state calculation. Middle: PMC simulation. Bottom: PMC with 10× multiplier on the scattering rate.

PMC simulations yield information about the distribution of the heat flux throughout the system. As the MD simulations do not lend themselves to the effective collection of heat flux data for long simulation times, we have opted to compare only the simple classical solution of the Fourier equation for heat flow with the PMC results. Figure 6 shows three vector plots, one showing the heat flux in the Fourier case and the others showing the heat flux in the PMC simulations for the standard scattering rate and the simulations with the scattering rate increased by a factor of 10. Note that the flux vectors have been normalized by the mean flux through each individual system, and the colour scale has been set to encompass the maximum and minimum values of all three systems, to enable effective comparison.

Perhaps unsurprisingly, there are no significant variations in the direction of the flux through the systems. In the colour detail one finds notable differences between the three systems in the areas of the bends and knee. Generally, the heat flux is not uniform throughout the systems. In the Fourier heat flux vector plot, we see an expected result, namely a fairly smoothly varying heat flux throughout the system, with strong gradients near the corners. The corner cutting effect is present here: Higher flux regions can be seen along the short path, or near the inside corners. In the centre plot, we see the heat flux in the PMC simulations. It shares similarities with the classical Fourier solution, where there seems to be more heat flowing through the inside corners and consequently less through the outside corners, though the effect seems overall to be subtler. The increased scattering rate plot yields a result closer to the Fourier result, with small bright and dark spots occurring more similarly to the previous scenario. In the 10× scattering plot, we note that the flux in tight corners is subject to noise, and that the heat flux is overall less smoothly varying than the other systems.

**Figure 7:**
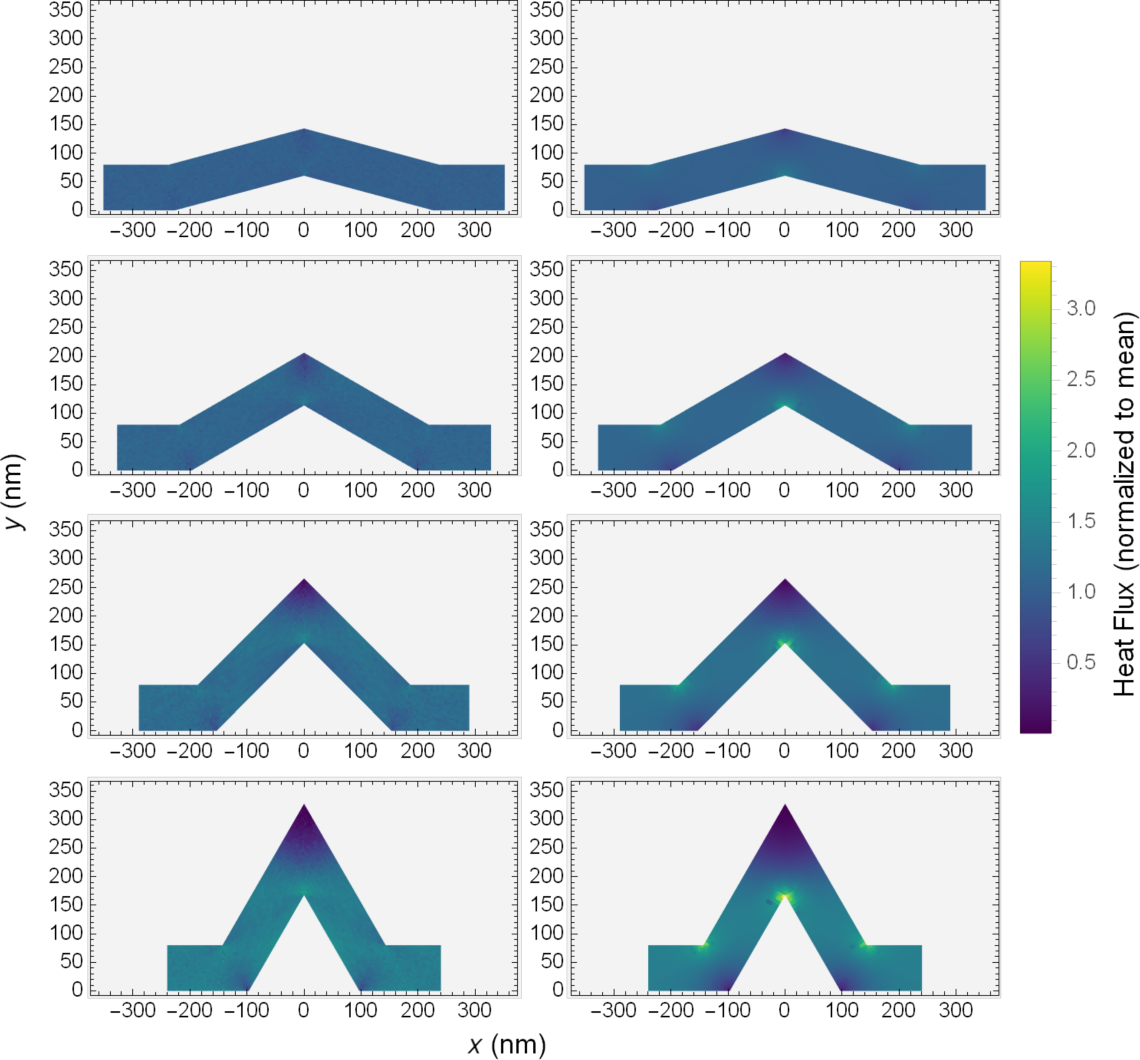
Colour maps of heat flux for PMC simulation (left column) and Fourier equation solution (right column) in kinked wires with kink angles of 15°, 30°, 45°, and 60°. The colour mapping indicates the value of the heat flux, normalized by the mean flux, through the system. The flux is unitless.

Looking to a more easily assessed visualization, namely a colour map, distinct kinds of behaviour can be seen in the PMC result when compared to the Fourier solution. Figure 7 shows colour maps of the (normalized) magnitude of the heat flux in PMC and Fourier systems with standard scattering rate, shown for four kink angles. The normalization here is again by the average flux through the system. The differences of colours in these figures allow us to examine the variation of heat flux within an individual system. A first examination of the effects of the kink angle on the distribution of the heat flux shows two principal differences between the two methods.

The first difference between the two is in the high (low) flux in the inside (outside) corners of the bends and knee. In the solutions of the Fourier equation, effects of the kink are localized. Far from the knee and bends, the heat flux is slowly varying. In the regions of the knee and bends, the variation of the flux is significant, showing clear high and low flux regions. Increasing the kink angle increases the size and intensity of the regions where the flux is non-uniform. There is an increase in the size of both high and low flux areas, a manifestation of the corner cutting effect.

Conversely, in the PMC result, the behaviour with kink angle seems to follow a similar trend, though the size and intensity of the high and low flux regions are much less. The corner cutting here appears to be lesser, as this would seem to be an effect that is more significant in diffusive scenarios. Phonons do not have a null mean free path and as such can be thought of as overshooting the optimal corner cut path. For the high flux inside corners, some phonons will not scatter and will continue past, while for the low flux areas, unscattered phonons may continue their trajectory there. This has the effect of muting the difference between the high and low flux regions. Essentially, while increasing the kink angle seems to promote corner cutting, the ballistic behaviour in the PMC systems moderates its intensity.

The second difference is seen by looking at the behaviour in the angled segments of the wires. As one would expect from a classical solution with continuous derivatives, the Fourier results show smoothly varying flux along the angled segments, while in the PMC simulation the overall flux seems to be much more homogeneous.

**Figure 8:**
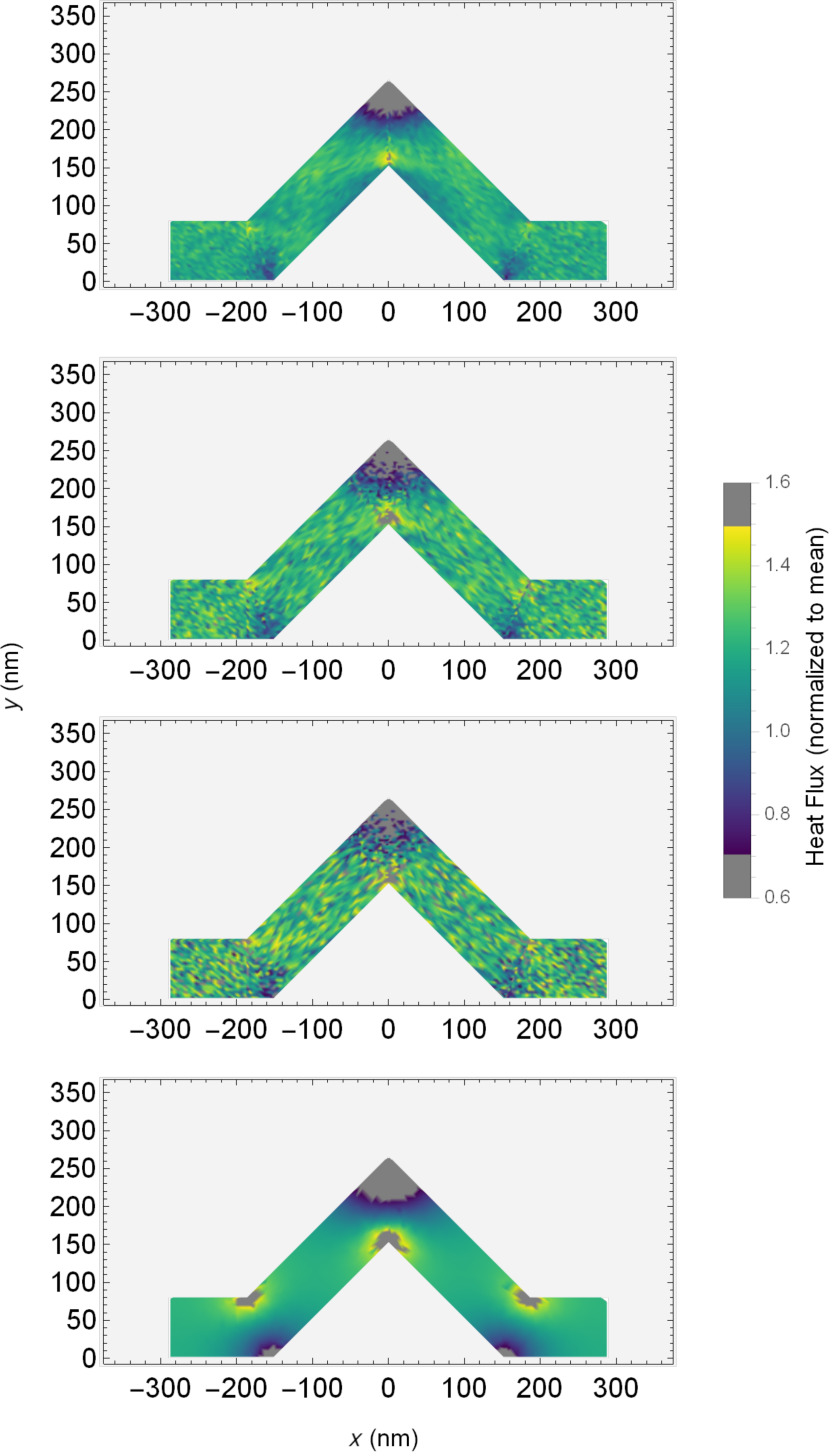
Clipped colour maps of normalized heat flux for the 45° system using (from top to bottom) PMC, PMC with 5× scattering, PMC with 10× scattering, and Fourier solution. The colour bar shows values where the colour scale is applied and where it is set to grey to improve resolution.

A careful eye might also note some faint patterning in the colours of the PMC results. As the colour scale for Figure 7 spans a large range, we can look to a more convenient visualization in Figure 8. Here we have clipped the colour range, setting the extreme low and high values in the bends and knee corners to grey. This allows the colour scale to span the range in the angled segments with higher resolution at the cost of hiding the highest and lowest values. In so doing, a clear and unique phenomenon emerges in the PMC result for standard scattering rates. There seem to be bright bands and a shadowing effect in the flux of these systems when compared to the Fourier solution. The bright band begins just before the lower portion of the left angled segment. It deflects upward, and then again as it reaches the upper boundary of the system, it deflects horizontally through the knee. It then deflects again off of the upper surface before being reflected down to the lower, mirroring the opposite angled segment. This concentration of the heat flux, especially as it seems more significant with larger kink angles, might explain the reduced conductances seen in the PMC simulations in Figure 5 when compared to the solution of the Fourier equation.

In addition to the shadowing effects, the variation of the flux within the angled segments in the PMC solution appears to be predominantly due to random noise resulting from the simulation method. This is in contrast with the solution of the Fourier equation, which varies much more smoothly in the angled segments. This is further emphasized by the centre two colour maps for systems with increased scattering rate, which show an increase in the noise throughout PMC systems.

The presence of these coloured bands in the PMC result indicate that the flux through the system is concentrated into a channel narrower than the wire itself. Although the angle of the channel seems to be similar to the angle of specular reflection of the phonons, it may not be exactly this. Similar patterns can be seen in other angled PMC systems. This behaviour is perhaps better explained by examining the shadowed areas that create the channel. In this case, those areas, which are immediately around corners, are shadowed as phonons travel past them unscattered, leading to a channel between dark areas. Overshoot and corner cutting are important here, as they are in other works involving serpentine nanowires [22].

If we look again to Figure 8 and focus on the results with increased scattering rates, the reflected ballistic phonons become more evident. Increasing the scattering rate by a factor of ten reduces the mean free path of phonons accordingly. As such, few, if any, phonons travel far enough to be reflected in an optical fashion, and the heat flux profile resembles the classical case.

The previous discussion of heat maps has been for cases where the phonon reflections at surfaces in the PMC solution have perfect specularity. In light of the discussed shadowing effect, it becomes interesting to investigate different scenarios for reflection specularity, ranging from perfectly specular (100% specularity) to perfectly diffusive (0% specularity). Perfectly diffuse surfaces can somewhat mimic nanoscale systems with high surface roughness. Note the probability of specular reflection is constant here and not a function of the phonon wavelength or incidence angle, so specularities between 0% and 100% should be taken with this in mind. More sophisticated methods for calculating the probability of specularity and its wavelength dependence have been suggested [38,41–42] and may be interesting for an expansion of this work.

**Figure 9:**
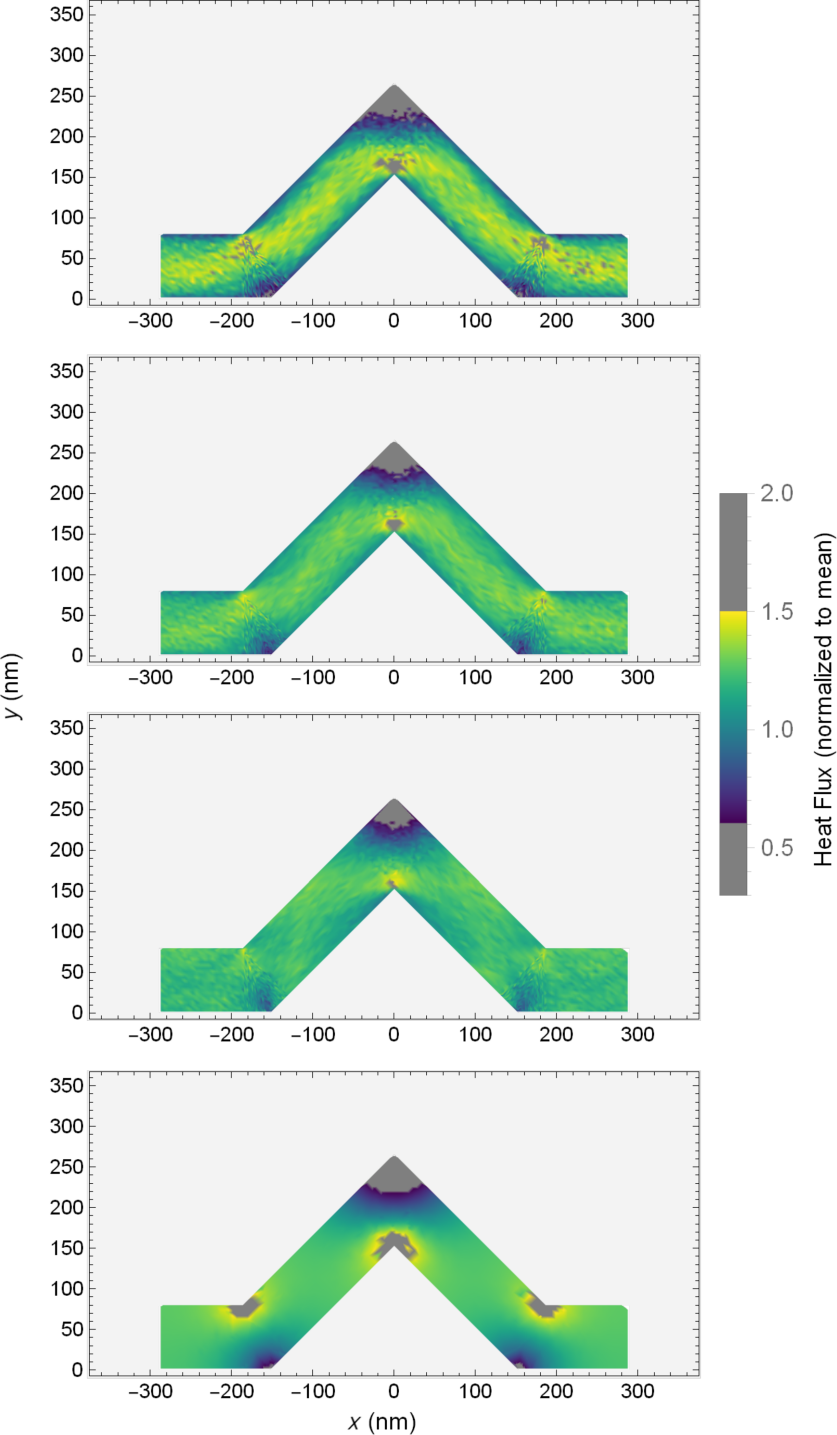
Clipped colour maps of normalized heat flux for 45° systems using (from top to bottom) PMC with 0% specularity, PMC with 50% specularity, PMC with 100% specularity and Fourier solution. The colour scale shows values where colour is clipped (set to grey) to improve resolution.

Figure 9 shows colour maps for the normalized flux for 45° kinked wires as solved using PMC (with varying degrees of specularity) and Fourier equation solutions. Colour scale clipping is again used to make the flow in intermediate regions more visible. In the uppermost graph, where the specularity is minimum, it is possible to see low flux regions along the sides of the wire, with a high flux band in the centre. This high flux band runs through the system from one end to the other, concentrating the flux in the middle of the wire and resulting in less flux near the edges. This is reminiscent of effects seen in labyrinthine nanowires [30]. As we increase the specularity, this effect remains to a certain degree, with still evidently lower flow along the surfaces of the wire and a concentration in the centre. At maximum specularity (the third graph), we return to the scenario seen in Figure 8, where reflections seem significant. In the perfectly specular case the flow reduction along the surfaces of the wire is no longer clearly visible at all points along the surface. Comparing all these PMC results with the Fourier result in the final graph of Figure 9, we do not see the same darkened edges but instead a smooth progression of the flux. The low flow region along the surface that can occur in PMC does not occur in the classical solution.

The tendency of flow to be reduced along the surface in low specularity systems seems reasonable, as the possibility of random reflection on contact with the surface would naturally lead to a reduction of flux in the direction of initial flow after reflection. Specular reflections result in less overall change of direction of the flux after reflection in contrast to diffuse reflections, which, in the limiting case, would result in flux being uniformly emitted in a semicircular region about the point of reflection.

Essentially, in the PMC model, the statement that the flux appears concentrated in a band narrower than the system dimensions seems further reinforced. Especially in low specularity cases, the flux of heat seems channelled in a narrow “river” through the wire itself, with lower flow along the surfaces of the system. This is somewhat reminiscent of frictional slowing of flow along a surface.

## Conclusion

In this work, we studied the effects of simple kinks of varying angles on thermal transport in simulated nanowires. The general trends of thermal conductance for low-dimensional systems are present here: nanowires exhibit reduced thermal conductance with increased length and increased thermal conductance with increased cross-sectional area [31–34]. As always, these can be among the most significant predictors of thermal transport. The length scaling seen here is similar to those seen in other works on serpentine nanowires [22], and the wires were not found to exhibit any non-monotonic anomalous behaviour [35].

In all systems investigated, the introduction of kinks with small bend angles produced a reduction in thermal conductance. This reduction is not necessarily monotonic. In the non-lattice-based simulations, the reduction of thermal conductance continued to much higher kink angles than in the lattice-based MD simulations.

MD simulations showed that around 20° of kink angle, the thermal conductance behaviour reversed, reaching a (local) maximum at 45°, corresponding to a [110] lattice orientation in the angled segments. Increased thermal conductivity along certain lattice directions has been seen in other nanosystems [38–39]. Beyond 45°, the thermal conductance decreases again.

In the case of the PMC simulations, the thermal conductance (for the standard scattering rate) decreases until about 50° of kink angle, after which it increases slightly. This may be related to symmetry angles, which minimise the number of reflections for ballistic phonons transiting through the system [40]. Fourier equation solutions scaled to match show an overall slower decrease of conductance with a minimum at a higher angle, though the two agree again around 60°.

All systems showed unstable results at very high kink angles, where anomalies in geometry greatly affect cross-sectional areas and, combined with the well-known corner cutting effect [22], necessitate very skeptical examination.

Overall, these results have strong implications for device design. When constructing nanodevices (single-crystalline ones in particular), one must be aware of the effects of bends and angles. Small bends seem to result in decreases in conductance overall, while the effects of larger bends depend on the nature of the system. When lattice effects dominate, lattice orientation must be taken into account. When ballistic phonon transport is significant, specularity and symmetry angles can be impactful.

Examining the heat flux in PMC simulations and comparing it to theoretical solutions of the heat equation is useful to understand which portions of a system dominantly influence thermal transport. For low kink angles, PMC and classical results generally agree. Phonon interaction with surfaces here is less than when the kink is large, leading to less reflective effects and long distances for phonons to travel uninterrupted. When kinks are introduced, and a careful investigation is done cleverly using colour scaling, it is possible to identify bands of heat flow through systems and the ever present corner cutting. This type of behaviour has been seen in various calculations for serpentine [22] and labyrinthine nanowires [30].

Corner cutting occurs more strongly in the diffusive heat equation solutions and in PMC results with artificially inflated scattering rates (e.g., more diffusive transport). In PMC results with low scattering rate, the heat flux shows some similarity to the behaviour of optical rays as it is reflected off the inside of the kink. Ray “phononics” (an analogue to ray optics) has been proposed as a tool for controlled heat flow in nanosystems [42].

An investigation of the effects of specularity in PMC results shows that systems with diffuse reflections tend to produce a “river-like” heat flow, while those with specular reflections result in a more “optics-like” banding effect.

The concentration of heat flux into areas smaller than the wire itself has significant implications for device design. Areas with more significant heat flow can amplify the effects of small changes in the wire. This is particularly true when impurities or defects are a concern [24]. Introduction of a source of scattering should produce different results if the flux of heat near the scattering source is large or small. It can even lead to a relative increase in thermal conductance depending on the position [15]. Understanding the flux could allow a device manufacturer to strategically place inclusions to modify thermal transport in a desired fashion.

Further investigations into variably kinked nanowires may include a study of temperature dependence. It is known that in serpentine nanowires [22] at higher temperatures ballistic behaviour is reduced. It is likely that variably kinked nanowires will show stronger kink effects below room temperature.

## Methodology

### Molecular dynamics

For this work, a Lennard-Jones potential [43–46] was used to simulate a solid with a face-centred cubic lattice in the well-known LAMMPS simulation framework [47]. The Lennard-Jones parameters σ and ε, effectively the length and energy scales, were set to 1, and a cut-off for the potential was set at 2.5σ. The set value of 1 for ε and σ fixes us to dimensionless units. Systems have an equilibrium temperature of 0.005, and they are under a thermostat at each end, for a temperature difference between the two of 0.001. Systems are simulated using open boundary conditions, that is, there is no use of periodic boundary conditions for these systems.

**Figure 10:**
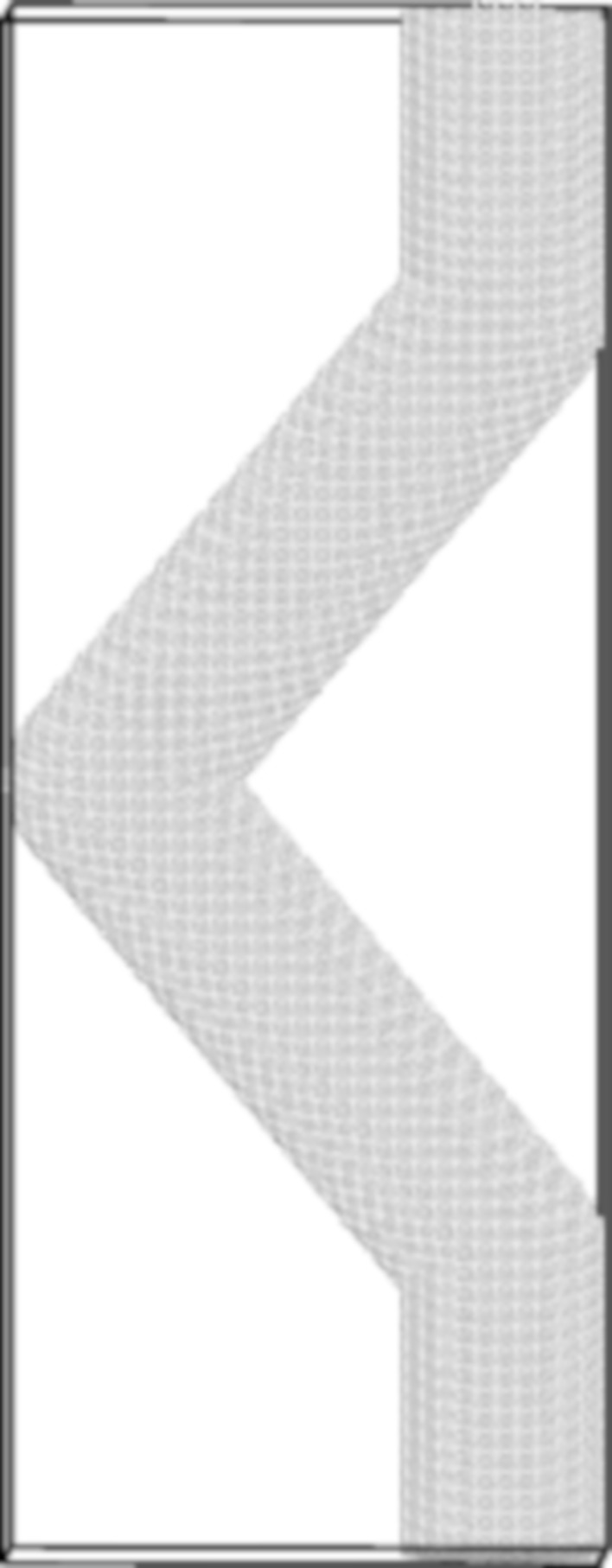
Image of the MD system for a wire with a kink angle of 40°. Visualization via OVITO [48].

The kinked wires used in MD are shaped from a single crystal oriented so that the straight portions follow along the [100] direction. These systems are build from cylindrical wires of radius *r*, and with fundamental segment length *l*, very similar to what is described in Figure 1. The thermostats occupy one third of the straight segments at either end. A spherical ball joint is used to join the wires at the bends and the knee, not shown in the aforementioned schematic. They are centred at the central joining point at the end of both cylinders. These joints are used to yield a more stable equilibration process, smoothing out sharp corners. Harsh angles can result in sintering during simulation, which can deform the wire, and must be handled carefully by observing the results of the various steps of preparation for the system. A visualization of a typical MD system can be see in Figure 10.

The use of a single [100] crystal for construction leads to different effective heat transport directions in the angled segment, depending on the kink angle of the wire. Additional unkinked systems where the lattice itself is oriented along the directions equivalent to those in the angled segments were constructed with dimensions to match the straight, unkinked wire (the 0° case).

In order to properly examine the kinked wire systems, a straight wire was first simulated at the equilibrium temperature for a lengthy period, and the effective lattice parameter at simulation temperature was calculated by examining the system dimensions. This was used as the lattice parameter for the kinked wires. The kinked wires then also underwent a lengthy equilibration process to allow them to expand as needed and remove any rotations about the centre of mass. This careful and extensive process allows us to create systems that avoid sintering at the inside of the knee, even for steep kink angles.

### Phonon Monte Carlo

The Boltzmann transport equation [49] is an excellent method for the description of phonon-based heat transport in nanoscale systems. Phonon Monte Carlo is a method that makes it possible to find solutions of the Boltzmann transport equation even for systems with complicated geometries. The first PMC simulations were published by Mazumder and Majumder [29] based on a preliminary work by Peterson [50]. The method was then further developed in the works by Lacroix et al. [27] and Péraud and Hadjiconstantinou [26,51]. PMC simulations simulate the propagation of packets of phonons through a system while utilizing stochastic methods to simulate the effect of scattering processes. Our implementation of the method closely follows the previous works [26–27,29,51]. Details of the implementation are given in [52].

Carrying out PMC simulations requires a model for the dispersion relations and scattering rates of the phonons in the system. In our work, we are using the parameters for silicon given by Jean et al. [28] in their work on simulations of nanoporous silicon and germanium. In that work, an isotropic model based on a parabolic fit is used for the description of the dispersion relations of the acoustic phonons. Since we are not attempting to obtain exact quantitative data for a specific material, we do not expect the limitation to acoustic phonons to be problematic. For the scattering rates of the phonons, Jean et al. [28] use a parametrization based on the formulas developed by Holland [53]. The values given in [28] correspond to the 1× scattering rate used in this work. Higher scattering rates, such as 10×, merely involve multiplying the rates by a constant factor. No impurity scattering was assumed in this work, and all simulations were carried out at a mid-point temperature of 300 K with a temperature difference of 20 K between the hot and cold ends of the system.

When a phonon packet interacts with a surface in a PMC simulation, the phonons scatter in one of two modes, namely diffusive or specular. Similar to previous works [27,29,54], our implementation of the PMC simulation method assumes a constant probability for specular reflection that is independent from the phonon wave length and incidence angle [52]. In order to obtain quantitative results, a more sophisticated approach such as Soffer’s equation [41] might be necessary. In this work, however, our goal was to show how an increase of diffusive reflection modifies the flow patterns observed at perfectly clean surfaces with 100% specularity. We believe that for this purpose a constant probability is sufficient.

The 2D geometry of the PMC simulations is a polygon that mimics the proportions of the corresponding MD system. The third dimension is treated as infinite in depth in the PMC simulations. This is equivalent to a system of finite depth but with perfectly specular surfaces perpendicular to that direction. This polygon takes into account any variations in width at the joins that occur from the joints used in the MD simulation. The supplementary sketches in Figure 2 illustrate this.

### Classical heat transport solutions

Fourier solutions were obtained through the use of Mathematica’s standard heat transfer library [55], built on a geometry matching that of the PMC, with appropriate boundary conditions to match the isothermal regions implemented in the PMC. Dimensionless units are used for these theoretical calculations.

### Weighted conductance estimate

The conductance estimate used in Figure 4 calculates the conductance by constructing a theoretical straight wire from the conductance of a 0° wire and the conductance of a wire where the lattice is oriented matching the angle of the angled segments. Building a wire out of these new components, it is possible to determine the weighting by treating all the segments as resistors in series. The new conductance *C*_a_(θ) estimate is then:


[3] $$C_{\text{a}}\left(\theta \right)={\left(\frac{1}{l_{0}+l_{\theta }}\left[\frac{l_{0}}{C_{0}}+\frac{l_{\theta }}{C_{\theta }}\right]\right)}^{-1},$$

where *l*_0_ and *l*_θ_ are the total lengths of the straight and angled segments of the wire, respectively, *C*_0_ is the conductance of a straight [100] wire, and *C*_θ_ is the conductance of a straight wire whose lattice is oriented along an angle θ with respect to the [100] wire.
